# Comprehensive proteomic analysis reveals SPRR3 as an early predictive biomarker for postoperative recurrence in pediatric chronic rhinosinusitis with nasal polyps^☆^

**DOI:** 10.1016/j.waojou.2026.101414

**Published:** 2026-06-28

**Authors:** Liyan Ni, Ruoqi Li, Siwen Xia, Xiaoqiong Wang, Bo Zheng, Yufeng Ye, Xuejun Liu

**Affiliations:** Department of Otolaryngology-Head and Neck Surgery, The Second Affiliated Hospital and Yuying Children's Hospital of Wenzhou Medical University, Wenzhou, Zhejiang Province, China

**Keywords:** Chronic rhinosinusitis with nasal polyps, Pediatrics, Proteomics, Recurrence, SPRR3, Biomarker

## Abstract

**Background:**

Pediatric chronic rhinosinusitis with nasal polyps (CRSwNP) exhibits a high rate of postoperative recurrence, yet effective molecular predictors remain lacking. This study aims to explore recurrence-associated proteins and evaluate their predictive value.

**Methods:**

Proteomic analysis was performed on nasal mucosa from a discovery cohort, with >1 year of follow-up to identify differentially expressed proteins (DEPs) associated with postoperative recurrence. Candidate proteins were validated in an independent cohort using western blotting, immunofluorescence, qRT-PCR, and serum ELISA.

**Results:**

In the discovery cohort, 6 pediatric patients suffering postoperative recurrence were classified as rCRSwNP, and 10 as non-rCRSwNP. Proteomic analysis revealed distinct expression patterns among control, non-rCRSwNP, and rCRSwNP groups. Four candidate proteins (CERS4, PLEKHA6, RAB29, SPRR3) were identified by intersecting the top 50 DEPs. SPRR3 and RAB29 were significantly upregulated in rCRSwNP, primarily localized to the nasal epithelium, as confirmed by western blotting and immunofluorescence. qRT-PCR further validated their increase in the recurrence group. Notably, paired tissue samples showed increased SPRR3 expression at revision surgery compared to baseline, and ELISA revealed higher serum SPRR3 levels in recurrent cases, correlating with recurrence risk. ROC and Kaplan–Meier analyses demonstrated potential predictive value for serum SPRR3.

**Conclusion:**

Our study identified a recurrence-associated proteomic signature in pediatric CRSwNP, marked by selective epithelial upregulation of SPRR3. Elevated serum SPRR3 was strongly linked to postoperative recurrence risk, supporting its utility as a predictive biomarker for identifying children at higher risk of relapse.

## Introduction

Chronic rhinosinusitis with nasal polyps (CRSwNP) is a persistent inflammatory disorder of the sinonasal mucosa marked by heterogeneous histopathology and variable prognosis.^1^, ^2^, ^3^ CRSwNP also occurs in children, with the highest incidence reported around 7–10 years of age.^4^ Pediatric cases frequently coexist with allergic rhinitis and asthma, display complex histopathological patterns, and present with severe symptoms that substantially affect school performance and daily life.^5^^,^^6^ Current management is mainly pharmacologic, yet many children have inadequate symptom control.^6^^,^^7^ Despite the increasing utilization of functional endoscopic sinus surgery (FESS), postoperative recurrence rates remain high, with up to 40% of patients requiring revision surgery.^8^^,^^9^ Postoperative recurrence, which leads to persistent symptoms, suboptimal disease control, and reduced quality of life, arises from a multifactorial pathophysiology that remains incompletely understood.^10^^,^^11^ Therefore, early identification of children at high risk of recurrence is crucial for optimizing treatment.

The pathophysiology of pediatric CRSwNP is not fully understood. The disease is characterized by a T2-skewed immune response, including eosinophilic inflammation, epithelial barrier dysfunction, and altered local cytokine signaling.^12^^,^^13^ Genetic factors, such as variants in IL-4Rα, TSLP, and CFTR, may further influence susceptibility and disease severity.^14^^,^^15^ Compared with adults, children with CRSwNP exhibit higher rates of allergic comorbidities and distinct inflammatory profiles, highlighting the need for pediatric-specific studies.^14^^,^^16^ Despite growing interest in postoperative recurrence in pediatric CRSwNP, the underlying molecular mechanisms remain incompletely understood, and reliable prognostic biomarkers are lacking.^17^ Previous studies using histology, nasal microbiome profiling, metabolomics, and transcriptomics have provided valuable insights, but they often fail to capture protein-level alterations that directly regulate cellular behavior and tissue remodeling. Most research has focused on adults, 18, 19, 20 leaving pediatric CRSwNP underexplored. Therefore, there is an urgent need for pediatric-specific, mechanism-based biomarkers to improve individualized risk assessment and guide postoperative management. Previous studies have identified recurrence-associated proteins in peripheral blood, such as CD109, which reduces recurrence by inhibiting TGF-β1-induced epithelial mesenchymal transition.^21^ In contrast, tissue-based proteomic profiling of sinonasal mucosa-the primary site of disease activity-remains surprisingly underexplored. Given that local mucosal changes are most proximate to disease pathogenesis and recurrence, the lack of tissue proteomic studies represents a critical knowledge gap that limits our understanding of CRSwNP biology and hinders the development of targeted therapeutic strategies.

In this study, we performed proteomic profiling of nasal polyp tissues from pediatric CRSwNP patients stratified by postoperative outcomes, aiming to identify recurrence-associated protein signatures. Candidate biomarkers were further validated in an independent cohort to evaluate their predictive value for postoperative recurrence. These findings offer novel insights into the molecular mechanisms underlying pediatric CRSwNP recurrence and may facilitate the development of targeted strategies for early risk stratification and personalized therapy.

## Methods

### Study design and patient selection

Two independent cohorts of pediatric CRSwNP patients undergoing functional endoscopic sinus surgery (FESS) and patients with benign skull base neoplasm (controls) were retrospectively included from our department between January 1, 2023 and December 1, 2023. The discovery cohort included 16 pediatric CRSwNP patients and 8 controls, while the validation cohort consisted of 60 pediatric CRSwNP patients and 30 controls. Patients were included if they met the following criteria: (1) diagnosis of CRSwNP according to the EPOS 2020 criteria,^22^ defined as ≥2 sinonasal symptoms persisting for ≥12 weeks with endoscopic confirmation of bilateral nasal polyps; (2) age younger than 18 years; (3) undergoing FESS for CRSwNP; and (4) provision of written informed consent by the patients and their legal guardians. Strict exclusion criteria were applied, including: (1) ≥18; (2) recent use (within 1 month before surgery) of antibiotics, oral or intranasal corticosteroids, immunotherapy, or antiallergic medications; (3) antrochoanal polyps or cystic fibrosis; (4) co-exciting other inflammatory or autoimmune conditions. The inclusion criteria for the control group were as follows: (1) pediatric patients undergoing surgery for benign skull base lesions (eg, juvenile nasopharyngeal angiofibroma); (2) no history of chronic rhinosinusitis, allergic rhinitis, or other inflammatory airway diseases; and (3) no clinical, endoscopic, or radiologic evidence of sinus inflammation before surgery. Detailed demographic and clinical information were collected for all subjects, including sex, age, body mass index (BMI), comorbidities, and peripheral blood eosinophil counts and percentages.

### Follow-up and definition of postoperative recurrence

All pediatric CRSwNP patients underwent FESS followed by standard postoperative follow-up for over 1 year within the same clinical team to ensure data completeness. Postoperative care included daily nasal saline irrigation, antibiotic therapy, topical corticosteroids, and periodic endoscopic debridement. Nasal cavity debridement was performed at 2 weeks and again at 1 month after surgery to facilitate mucosal healing. Follow-up visits were scheduled at 3, 6, and 12 months postoperatively, with subsequent endoscopic evaluations every 6 months thereafter, as previously described.^23^, ^24^, ^25^ Postoperative recurrence was defined as the reappearance of nasal polyps confirmed by nasal endoscopy and/or CT imaging, accompanied by persistent clinical symptoms lasting >8 weeks despite standard medical therapy. When recurrent polyps were identified on endoscopic examination, patients received standard medical treatment and were re-evaluated after 8 weeks. If no improvement was observed, the date of the initial endoscopic detection was recorded as the time of recurrence.^23^^,^^24^ Based on follow-up outcomes, patients were classified into recurrence CRSwNP (rCRSwNP) and non-recurrence CRSwNP (non-rCRSwNP) groups.

### Proteomic profiling and data analysis

Freshly resected nasal polyps and middle turbinate tissues were snap-frozen in liquid nitrogen at the time of surgery. Samples were then cryopulverized and extracted in RIPA buffer (Beyotime, Shanghai, China) containing protease and phosphatase inhibitors to preserve protein integrity. After high-speed centrifugation at 12,000×*g* for 15 min at 4°C, the supernatants containing soluble proteins were collected. Protein concentrations were quantified using the bicinchoninic acid (BCA) assay kit (Beyotime, Shanghai, China) to normalize sample input. Equal amounts of protein from each sample were reduced, alkylated, and enzymatically digested with sequencing-grade modified trypsin according to the filter-aided sample preparation (FASP) protocol. Peptides were desalted on C18, vacuum-dried, and reconstituted in 0.1% formic acid. Samples were analyzed by LC–MS/MS on a high-resolution instrument in data-dependent acquisition to maximize coverage. Raw files were processed in MaxQuant for identification and label-free quantification against the human UniProt/SwissProt database. Proteins present in ≥70% of samples within each group were retained. Differential expression was assessed in Perseus using p < 0.05 and fold change >1.5 as previously described.^26^

### Western blotting (WB) analysis

Tissue samples were homogenized in RIPA lysis buffer supplemented with protease and phosphatase inhibitors. Following centrifugation at 12,000×*g* for 15 min at 4°C, the supernatants were collected, and protein concentrations were measured using a BCA protein assay kit (NCM, Changzhou, China). Equal amounts of total protein (10 μg per lane) were separated by SDS-PAGE and transferred onto PVDF membranes. Membranes were blocked with 5% skim milk for 1 h at room temperature and incubated overnight at 4°C with primary antibodies against CERS4 (1:2000, Invitrogen, Carlsbad, USA), PLEKHA6 (1:1000, Invitrogen, Carlsbad, USA), RAB29 (1:1000, Cell Signaling Technology, Danvers, USA), and SPRR3 (1:1000, Proteintech Group, Wuhan, China), as well as GAPDH (1:2000, Proteintech Group, Wuhan, China) as the internal control. After washing, membranes were incubated with appropriate HRP-conjugated secondary antibodies for 1 h at room temperature. Protein bands were visualized using an enhanced chemiluminescence (ECL) detection system, and band intensities were quantified using ImageJ software (NIH, Bethesda, USA) and normalized to GAPDH levels.

### Quantitative reverse transcription polymerase chain reaction (qRT-PCR)

Total RNA was isolated from frozen nasal tissue using TRIzol reagent (Invitrogen, Carlsbad, USA) following the manufacturer's protocol. RNA concentration and purity were assessed with a NanoDrop spectrophotometer (Thermo Fisher Scientific). One microgram of total RNA was reverse-transcribed into cDNA using the PrimeScript RT Reagent Kit (Takara, Dalian, China). Quantitative PCR was then performed using SYBR Premix Ex Taq (Takara) on a real-time PCR system. Relative gene expression levels were calculated using the 2^−ΔΔCt^ method, with GAPDH serving as the internal reference for normalization. Primer sequences are listed in Table S1.

### Immunofluorescence staining

Paraffin sections (5 μm) were prepared from 4% paraformaldehyde-fixed tissues. Slides were baked at 60°C for 2 h, deparaffinized in xylene, rehydrated through graded ethanol, and rinsed in water. Antigen retrieval was then performed by heating in citrate buffer (microwave, 20 min), followed by cooling and PBS washes. Sections were permeabilized with 0.1% Triton X-100 and blocked with 5% bovine serum albumin (BSA) for 1 h at room temperature. They were then incubated overnight at 4°C with primary antibodies against CERS4 (1:200, Invitrogen, Carlsbad, USA), PLEKHA6 (1:200, Invitrogen, Carlsbad, USA), RAB29 (1:200, Cell Signaling Technology, Danvers, USA), and SPRR3 (1:200, Proteintech Group, Wuhan, China). After washes, sections were incubated at room temperature for 1 h in the dark with fluorophore-conjugated secondary antibodies. Nuclei were counterstained with DAPI, and slides were mounted in antifade medium. Images were acquired on a fluorescence or confocal microscope, and signal intensity was quantified in ImageJ (NIH, Bethesda, USA).

### Enzyme-linked immunosorbent assay

Peripheral blood samples were collected and centrifuged at 3000×*g* for 10 min to obtain serum, which was stored at −80°C until analysis. Serum SPRR3 concentrations were measured using a commercially available enzyme-linked immunosorbent assay (ELISA) kit (MyBioSource, San Diego, USA) according to the manufacturer's instructions. Briefly, serum samples and standards were added to 96-well plates pre-coated with SPRR3-specific antibodies and incubated at 37°C for 1–2 h. After washing, wells were incubated with a biotinylated detection antibody followed by HRP-conjugated streptavidin. Colorimetric development was achieved by adding tetramethylbenzidine (TMB) substrate, and the reaction was stopped with sulfuric acid. Optical density (OD) values were measured at 450 nm using a microplate reader (Model, Company). SPRR3 concentrations were calculated from a standard curve and expressed as ng/mL.

### Statistical analysis

Data were analyzed in SPSS 26 (IBM, Armonk, NY, USA) and GraphPad Prism 8 (GraphPad, San Diego, CA, USA). Categorical variables were compared with the chi-square or Fisher's exact test. Continuous data are presented as mean ± SEM. Group differences were evaluated with the Kruskal–Wallis H test, with two-tailed Mann–Whitney U tests for post-hoc pairwise comparisons. The predictive performance of candidate protein levels for CRSwNP recurrence was evaluated by constructing receiver operating characteristic (ROC) curves and calculating the area under the curve (AUC). Kaplan–Meier survival curves were generated to analyze the time to recurrence, and differences between groups were assessed using the log-rank test. A p-value < 0.05 was considered statistically significant.

## Result

### Proteomic analysis identifies tissue protein expression profiles associated with postoperative recurrence in pediatric CRSwNP

**Fig. 1:**
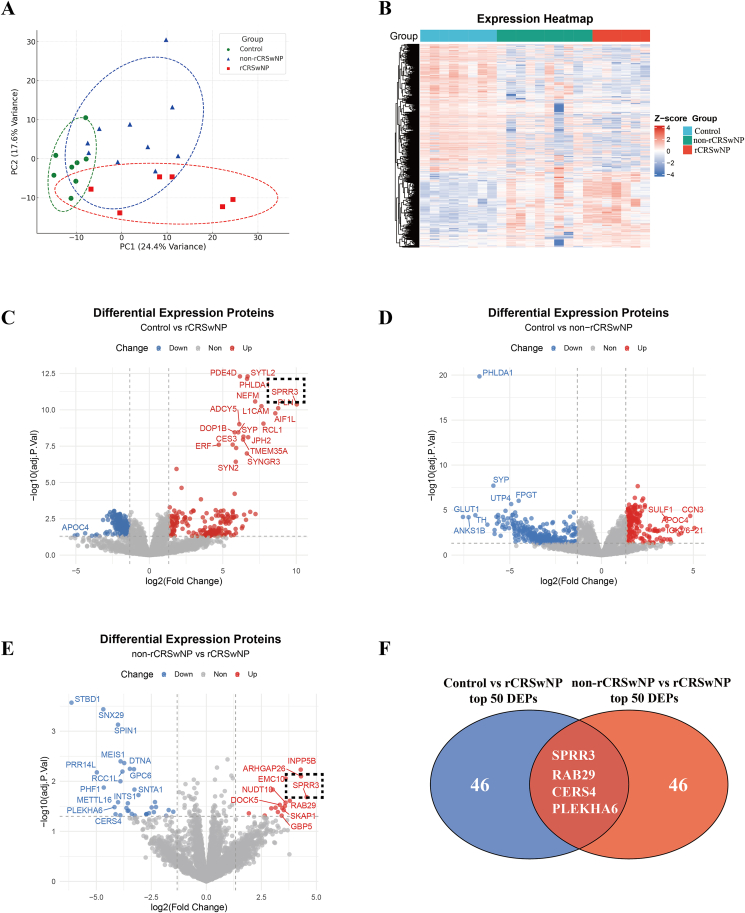
**Proteomic profiling of nasal polyp tissues reveals recurrence-associated protein expression patterns in pediatric CRSwNP**. (A) Principal component analysis of proteomic profiles. (B) Heatmap displaying the DEPs among the 3 groups. (C–E) Volcano plots highlighting DEPs between each comparison group. (F) Venn diagram of the top 50 DEPs from each group comparison reveals 4 overlapping proteins. CRSwNP, chronic rhinosinusitis with nasal polyps; DEPs, differentially expressed proteins

In the discovery cohort, after 1 year of postoperative follow-up, 6 pediatric CRSwNP patients were classified into the rCRSwNP group, while the remaining 10 patients were assigned to the non-rCRSwNP group. The clinical characteristics of all subjects are summarized in Table S2. Principal component analysis (PCA) showed clear separation among controls, non-rCRSwNP, and rCRSwNP, indicating substantial differences in global protein expression profiles. Heatmap analysis further revealed distinct molecular protein signatures in nasal polyp tissues from rCRSwNP children compared with non-rCRSwNP and controls. Volcano plots identified significantly dysregulated proteins in rCRSwNP vs. controls (Fig. 1C), non-rCRSwNP vs. controls (Fig. 1D), and rCRSwNP vs. non-rCRSwNP (Fig. 1E). To explore the potential association of these DEPs with postoperative recurrence risk, the top 50 DEPs from each comparison were intersected using a Venn diagram. This cross-comparison identified 4 core candidate proteins-CERS4, PLEKHA6, RAB29, and SPRR3-that were shared across analyses (Fig. 1F), suggesting a close association with postoperative recurrence of CRSwNP.

### Elevated SPRR3 correlates with postoperative recurrence risk in CRSwNP

**Fig. 2:**
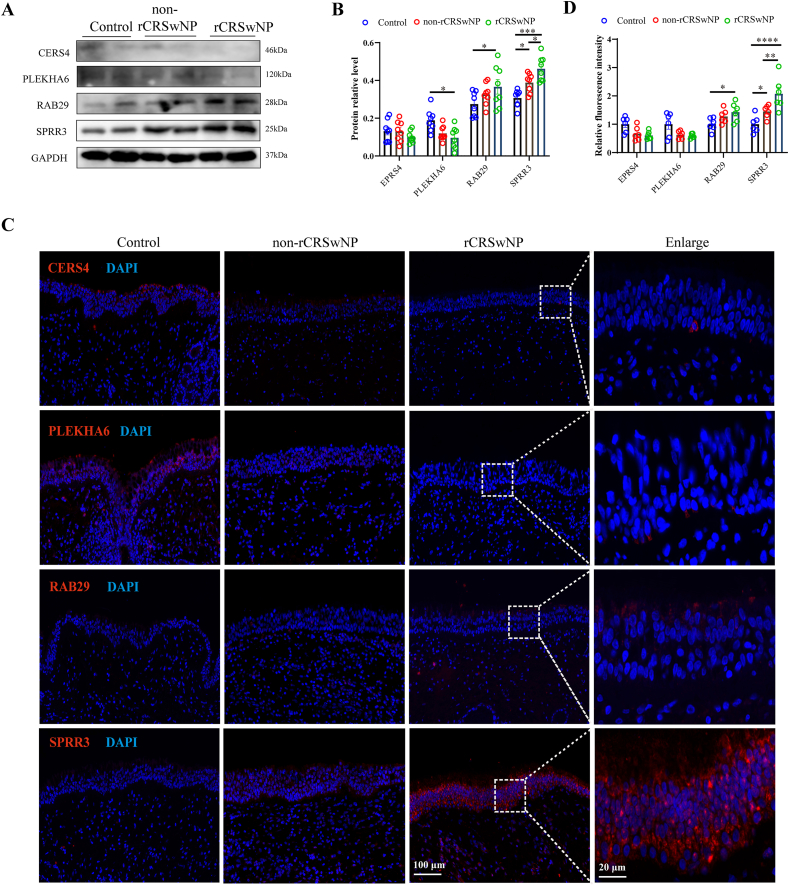
**Validation of candidate DEPs in an independent cohort**. (A-B) WB analysis of candidate DEPs expression among the 3 groups (n = 12). (C–D) Immunofluorescence was performed to examine the expression and localization of 4 DEPs among the 3 groups (n = 6). **∗**P < 0.05; **∗∗**P < 0.01, **∗∗∗**P < 0.001, **∗∗∗∗**P < 0.0001. CRSwNP, chronic rhinosinusitis with nasal polyps; DEPs, differentially expressed proteins; WB, western blotting

To further validate the expression of the 4 candidate proteins, a validation cohort of 23 rCRSwNP children and 37 non-rCRSwNP children after 1 year of follow-up was analyzed. The clinical characteristics of this cohort are shown in Table 1. WB analysis (Fig. 2A and B) revealed that SPRR3 protein levels in the recurrent group were upregulated compared with both the control (0.5 ± 0.1 vs 0.3 ± 0.1, P < 0.001) and non-rCRSwNP groups (0.5 ± 0.1 vs 0.4 ± 0.1, P = 0.034). Immunofluorescence staining further confirmed stronger SPRR3-specific signals in the nasal polyp epithelium of the recurrent group, with higher expression intensity than that observed in the control (2.1 ± 0.6 vs 1.0 ± 0.3, P < 0.001) and non-rCRSwNP groups (2.1 ± 0.6 vs 1.5 ± 0.2, P = 0.002) (Fig. 2C and D). These findings indicate that SPRR3 is specifically overexpressed in the nasal epithelium of CRSwNP patients with recurrence and may play a role in the pathophysiology of postoperative recurrence.

**Table 1:** Clinical characteristic in validation cohort.

Variable	Control	non-rCRSwNP	rCRSwNP	P
Gender, male/female,	18/12	21/16	12/11	0.383
Age, year	14.4 ± 3.3	14.2 ± 3.0	13.8 ± 2.7	0.870
BMI, kg/m^2^	18.3 ± 1.5	18.7 ± 1.1	18.9 ± 0.9	0.798
Allergic rhinitis, Yes/No	0/30	6/31	9/14	<0.001
Asthma, Yes/No	0/30	5/32	6/17	0.003
Blood eosinophil count, × 10^9^/L	0.05 ± 0.04	0.13 ± 0.08	0.27 ± 0.16	<0.001
Blood eosinophil percentage, %	1.8 ± 1.1	2.6 ± 1.5	3.4 ± 2.0	0.005

CRSwNP, chronic rhinosinusitis with nasal polyps; BMI, body mass index

**Fig. 3:**
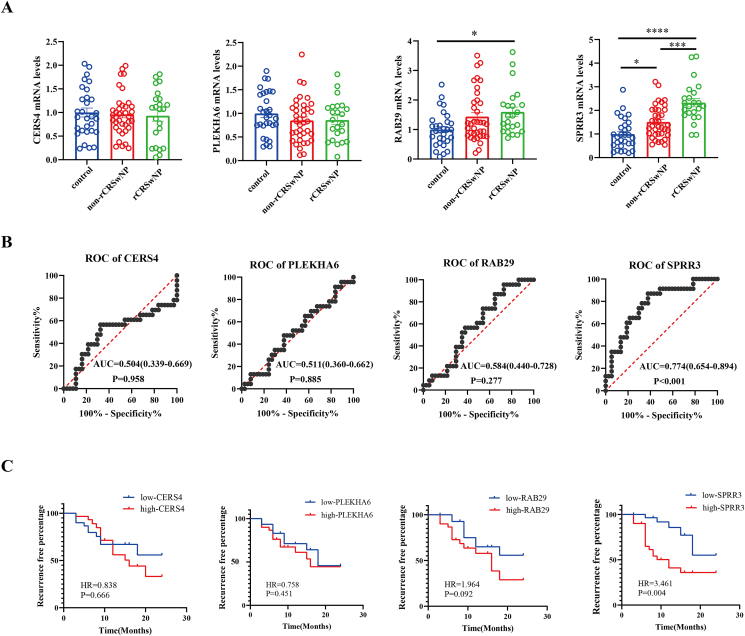
**Tissue SPRR3 expression correlates with postoperative recurrence risk in pediatric CRSwNP**. (A) qRT-PCR was performed to evaluate the mRNA expression of 4 DEPs. (B) ROC curves were performed to assess the predictive values of 4 indicators for postoperative recurrence in CRSwNP patients. (C) Kaplan–Meier survival analysis showing the association between tissue expression of 4 DEPs and recurrence risk. **∗**P < 0.05; **∗∗**P < 0.01, **∗∗∗**P < 0.001, **∗∗∗∗**P < 0.0001. CRSwNP, chronic rhinosinusitis with nasal polyps; ROC, receiver operating characteristic; DEPs, differentially expressed proteins

**Fig. 4:**
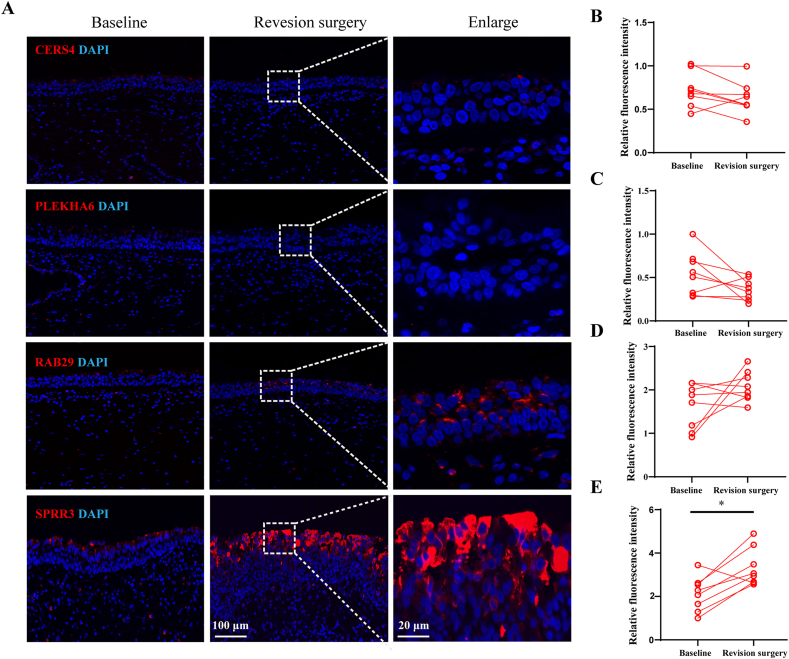
**Increased tissue SPRR3 expression during postoperative recurrence in the same CRSwNP children**. (A) Representative immunofluorescence images showing the fluorescence expression of the 4 DEPs in recurrent samples compared with their baseline counterparts. (B-E) Comparison of fluorescence intensity of the 4 DEPs in nasal polyp tissues before and after recurrence in the same patients. ∗P < 0.05, ∗∗P < 0.01. CRSwNP, chronic rhinosinusitis with nasal polyps; DEPs, differentially expressed proteins

To further confirm the association between these 4 DEPs and postoperative recurrence risk, we expanded the sample size for qRT-PCR analysis. As shown in Fig. 3A, SPRR3 mRNA expression was higher in the rCRSwNP group compared with the control (2.3 ± 0.9 vs 1.0 ± 0.6, P < 0.0001) and non-rCRSwNP groups (2.3 ± 0.9 vs 1.5 ± 0.7, P < 0.001)). ROC curve analysis demonstrated that SPRR3 mRNA had the highest predictive value for postoperative recurrence among the 4 candidate markers (Fig. 3B). Furthermore, Kaplan–Meier survival analysis revealed that CRSwNP patients with elevated SPRR3 mRNA levels were higher risk of postoperative recurrence (HR = 3.58, P = 0.002) (Fig. 3C). These findings suggest that aberrant SPRR3 expression in nasal tissue may play a role in the pathophysiological mechanisms underlying postoperative recurrence of pediatric CRSwNP. Interestingly, we compared SPRR3 expression in paired specimens obtained from the primary and revision surgeries of 8 pediatric CRSwNP patients. Immunofluorescence analysis revealed that tissue SPRR3 levels were higher in recurrent samples than in their baseline counterparts (2.1 ± 0.8 vs 3.3 ± 0.9, P = 0.011, Fig. 4). These findings further support the potential of SPRR3 as a molecular marker for postoperative recurrence in CRSwNP.

**Fig. 5:**
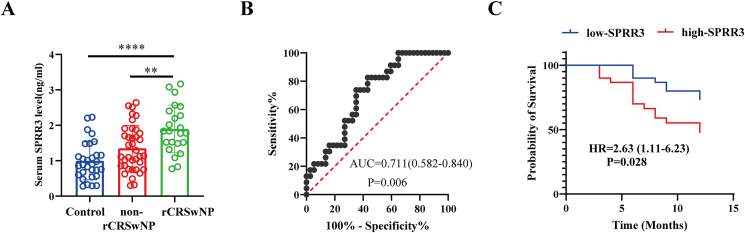
**Serum SPRR3 level is associated with postoperative recurrence in pediatric CRSwNP patients**. (A) ELISA showing the serum SPRR3 concentration among the 3 groups. (B) ROC curves revealing the predictive values of serum SPRR3 for postoperative recurrence in pediatric CRSwNP patients. (C) Kaplan–Meier survival analysis showing the association between serum SPRR3 level and postoperative recurrence risk. **∗∗**P < 0.01, **∗∗∗**P < 0.001. CRSwNP, chronic rhinosinusitis with nasal polyps; ROC, receiver operating characteristic

### Circulating SPRR3 is a potential biomarker for predicting postoperative recurrence in CRSwNP

Exploring peripheral blood biomarkers to early predict the risk of postoperative recurrence in pediatric CRSwNP is a current research focus with important clinical implications. To determine whether serum SPRR3 levels are associated with postoperative recurrence risk, we measured serum SPRR3 concentrations using ELISA. The results showed that serum SPRR3 levels were higher in the rCRSwNP group compared with the control (4.3 ± 1.6 vs 2.5 ± 1.1, P < 0.001) and non-rCRSwNP groups (4.3 ± 1.6 vs 3.0 ± 1.7, P = 0.006) (Fig. 5A). Furthermore, serum SPRR3 levels were positively correlated with SPRR3 expression in nasal polyp tissues (r = 0.411, P = 0.001), as shown in Figure S1, suggesting that circulating SPRR3 may partially reflect local tissue expression. ROC curve and Kaplan–Meier survival analyses further revealed a strong association between serum SPRR3 levels and the risk of postoperative recurrence (HR = 2.63, P = 0.028) (Fig. 5B-C). The diagnostic performance of serum SPRR3, including sensitivity, specificity, and predictive values at the optimal cutoff, is presented in Table S3. These findings suggest that peripheral blood SPRR3 may serve as a potential molecular biomarker for predicting postoperative recurrence in pediatric CRSwNP.

## Discussion

In this study, we applied tissue proteomics to identify differentially expressed proteins associated with postoperative recurrence in pediatric CRSwNP and nominated SPRR3 as an early predictive biomarker. SPRR3 was significantly upregulated in nasal mucosa from recurrent cases and was predominantly localized to the nasal epithelium. Validation analyses showed that both tissue and serum SPRR3 provided good predictive performance for recurrence, with higher levels correlating with increased postoperative risk. These findings implicate SPRR3 in the pathogenesis of pediatric CRSwNP recurrence and support its use as an early biomarker for risk stratification and postoperative monitoring.

Small proline-rich protein 3 (SPRR3) belongs to the SPRR protein family, which is mainly expressed in epithelial tissues.^27^, ^28^, ^29^ Previous studies showed that SPRR3 was involved in the differentiation of epithelial cells, maintenance of barrier function, and modulation of damage response.^30^^,^^31^ Bae et al^32^ found that the PM2.5 induced upregulation of SPRR3 in skin keratinocytes, which inhibited cilia formation and leads to epithelial damage. In additional, Hu et al^33^ demonstrated that cigarette smoke could induce SPRR3 upregulation in human bronchial epithelial cells involved in pathologic changes in chronic obstructive pulmonary disease (COPD). Recent evidence also suggests that other SPRR family members, such as SPRR2A, is involved in mucosal inflammation and barrier dysfunction in CRSwNP.^34^ However, SPRR3 expression characteristics in CRSwNP and its relationship with postoperative recurrence have not been clarified. Given that epithelial barrier impairment and persistent inflammation are key pathological features of CRSwNP, it is plausible that SPRR3 may play a role in its pathogenesis. In our study, proteomic analysis and cohort validation revealed significantly elevated SPRR3 expression in recurrent CRSwNP compared to non-recurrent case and controls, with localization primarily to the nasal epithelium. These findings suggest that SPRR3 upregulation may reflect inflammation-driven epithelial barrier damage or impaired mucosal repair processes, and may be mechanistically involved in disease progression and recurrence.

Impaired epithelial barrier function, particularly tight junction disruption and defective mucosal repair, has been shown to facilitate persistent exposure to environmental allergens and pathogens, thereby sustaining chronic inflammation and increasing the risk of nasal polyp recurrence.^35^, ^36^, ^37^, ^38^ SPRR3, as a structural component of the cornified envelope, plays a critical role in epithelial differentiation and barrier integrity through crosslinking of envelope proteins.^39^, ^40^, ^41^ In our study, SPRR3 expression was significantly elevated in the recurrence group compared to non-recurrent CRSwNP children, as revealed by tissue proteomics and validated through multiple approaches. These findings suggest that SPRR3 may participate in disease recurrence by modulating epithelial responses to repeated mucosal injury. Notably, SPRR3 showed good sensitivity and specificity in predicting recurrence, and survival analysis further confirmed that high tissue expression of SPRR3 was associated with increased recurrence risk. Importantly, we also observed elevated SPRR3 levels in the serum of recurrent patients, which strongly correlated with the risk of postoperative recurrence, indicating its potential as a non-invasive biomarker for postoperative risk stratification. Together, these results suggest that SPRR3 overexpression may reflect a maladaptive or compensatory epithelial remodeling process in response to chronic inflammation and tissue damage, and may contribute to the pathogenesis and recurrence of CRSwNP.

This study has several limitations. First, the sample size was modest due to the single-center design and the relatively low incidence of pediatric CRSwNP, which may limit generalizability. Second, we validated differentially expressed proteins and assessed their clinical utility but did not conduct mechanistic experiments; moreover, only the top DEPs from the proteomic screen were validated, so other DEPs may also be biologically or clinically relevant. In addition, to strengthen the theoretical foundation of this research, future studies should integrate multi-omics and genetic analyses. Finally, the findings require confirmation in larger, multi-center cohorts with long-term follow-up to establish robustness and external validity.

## Conclusion

Our study identifies SPRR3 as a novel protein associated with postoperative recurrence in pediatric CRSwNP, showing consistently elevated expression in both nasal tissue and serum in recurrent cases. Given its predominant localization in the epithelium and its reported roles in epithelial barrier formation and inflammatory responses, SPRR3 may be involved in epithelial remodeling and barrier dysfunction during disease progression. The observed association between SPRR3 expression and recurrence risk suggests that it may serve as a potential biomarker for identifying children at increased risk of postoperative recurrence. Measurement of preoperative serum SPRR3 levels may help facilitate risk stratification and guide postoperative management. Children with higher SPRR3 levels may benefit from closer postoperative surveillance and optimized medical therapy to allow earlier detection and management of recurrence.

## Author contribution

(I) Conception and design: Liyan Ni and Xuejun Liu.

(II) Administrative support: Liyan Ni and Xuejun Liu.

(III) Collection and assembly of data: Xiaoqiong Wang, Siwen Xia, Bo Zheng, Yufeng Ye.

(IV) Histopathological experiments: Siwen Xia and Bo Zheng.

(V) Manuscript writing: All authors.

(VI) Final approval of manuscript: All authors.

## Ethics approval

This study was approved by the Human Ethics Committee of The Second Affiliated Hospital and Yuying Children's Hospital of Wenzhou Medical University (2025-k-326-01), and all participants provided written informed consent in accordance with the Declaration of Helsinki.

## Consent for publication

All authors have seen and approved the last version and agreed to the publication of the work.

## Disclosure statement

No generative AI and AI-assisted technologies were used. Nothing to disclose.

## Availability of data and material

All data generated or analyzed during this study are included in this published article and its Supplemental file. More related data of the current study are available from the corresponding author upon reasonable request.

## Funding source

This research was supported by 10.13039/501100007194Wenzhou Municipal Science and Technology Bureau (Grants Y20220170, Y20240326), Zhejiang Provincial Clinical Research Center for Pediatric Diseases (Grants ZJEK2310Y). Wenzhou Medical Association Scientific Research Project (Grants 202303KZ1).

## Declaration of competing interest

There are no patents, products in development, or marketed products to declare.

## Figures and Tables

**Figure:**
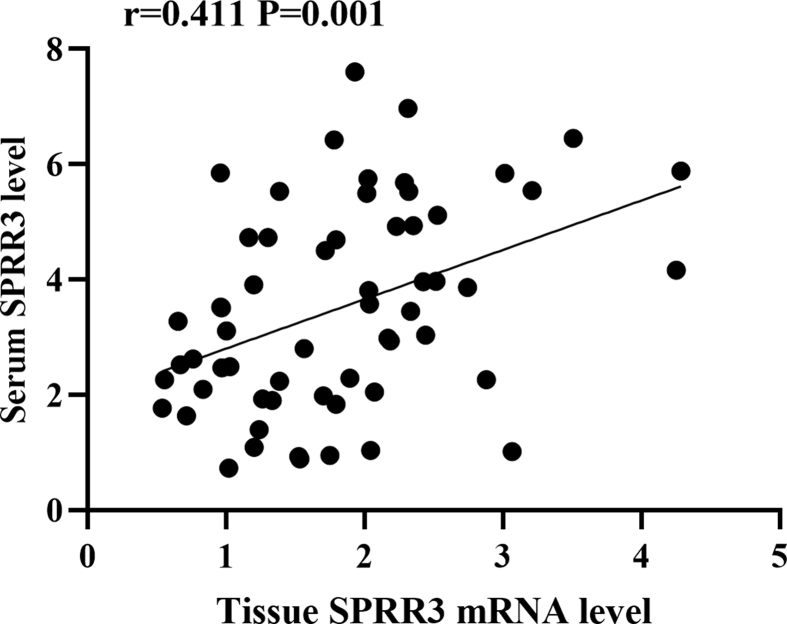
Figure S1. Correlation between serum SPRR3 levels and tissue SPRR3 mRNA expression in pediatric CRSwNP patients. CRSwNP, chronic rhinosinusitis with nasal polyps
