# Supplementary material for: Comprehensive proteomic analysis reveals SPRR3 as an early predictive biomarker for postoperative recurrence in pediatric chronic rhinosinusitis with nasal polyps
Source: World Allergy Organ J. 2026 Jun 28;19(8):101414. doi: 10.1016/j.waojou.2026.101414 (PMC13324294; doi:10.1016/j.waojou.2026.101414)
Supplement: Multimedia component 1 [file mmc1.docx]

| Gene | Forward primer | Reverse primer |
| --- | --- | --- |
| GAPDH | GGGTATATGGTAACCTTGTGTCCC | CCTTTGCAGGGCTGAGTCAG |
| CERS4 | GAGCCTGGGAGTGTTCATGG | GCCCATACATCCCCCTGAAC |
| PLEKHA6 | TTCCCTCAGCTTGGAAAGGC | TCCTGGTATGTTGTCACTTAGCC |
| RAB29 | TGCTTTCTCGTCAGAGCAGCCCA | TCGGGGATCGGGGGTCGCTCGTTTTAA |
| SPRR3 | TGAACCAGGCAGCATCAAGG | GGAAGAGGGTGTCCAGCATC |

Table S1. Primers sequences
