# Supplementary material for: Comprehensive proteomic analysis reveals SPRR3 as an early predictive biomarker for postoperative recurrence in pediatric chronic rhinosinusitis with nasal polyps
Source: World Allergy Organ J. 2026 Jun 28;19(8):101414. doi: 10.1016/j.waojou.2026.101414 (PMC13324294; doi:10.1016/j.waojou.2026.101414)
Supplement: Multimedia component 2 [file mmc2.docx]

| Variable | Control | non-rCRSwNP | rCRSwNP | P |
| --- | --- | --- | --- | --- |
| Gender, male/female, | 4/4 | 6/4 | 3/3 | 0.889 |
| Age, year | 15.8±2.9 | 14.4±3.6 | 14.8±3.2 | 0.768 |
| BMI, kg/m^2^ | 19.5±1.2 | 19.1±1.6 | 18.8±1.6 | 0.526 |
| Allergic rhinitis, Yes/No | 0/8 | 3/7 | 2/4 | 0.204 |
| Asthma, Yes/No | 0/8 | 1/9 | 2/4 | 0.167 |
| Blood eosinophil count, ×10^9^/L | 0.09±0.04 | 0.12±0.04 | 0.13±0.05 | 0.133 |
| Blood eosinophil percentage, % | 1.5±0.7 | 1.7±0.7 | 2.3±0.9 | 0.174 |

Table S2 Clinical characteristic in discover cohort

CRSwNP, chronic rhinosinusitis with nasal polyps; BMI, body mass index.
