# Supplementary material for: Comprehensive proteomic analysis reveals SPRR3 as an early predictive biomarker for postoperative recurrence in pediatric chronic rhinosinusitis with nasal polyps
Source: World Allergy Organ J. 2026 Jun 28;19(8):101414. doi: 10.1016/j.waojou.2026.101414 (PMC13324294; doi:10.1016/j.waojou.2026.101414)
Supplement: Multimedia component 3 [file mmc3.docx]

| Variable | Cut-off value | Sensitivity% | Specificity% | Likelihood ratio | P-value |
| --- | --- | --- | --- | --- | --- |
| Serum SPRR3 level | 2.869 | 82.61 | 56.76 | 1.910 | 0.006 |

Table S3 ROC analysis of serum SPRR3 for predicting postoperative recurrence

ROC: receiver operating characteristic
